# A Brain Connectivity Approach to Detect Diffusion-Weighted Imaging Changes in Post-Traumatic Epilepsy

**DOI:** 10.3390/bioengineering13060598

**Published:** 2026-05-22

**Authors:** Emanuele C. Amato, Claudia Giliberti, Nicola Amoroso, Kseniia Kriukova, Alfonso Monaco, Ester Pantaleo, Tommaso Maggipinto, Loredana Bellantuono, Antonio La Calamita, Roberto Bellotti, Paul M. Vespa, Dominique Duncan, Marianna La Rocca

**Affiliations:** 1 Dipartimento Interateneo di Fisica, Università degli Studi di Bari Aldo Moro, 70125 Bari, Italy; c.giliberti3@studenti.uniba.it (C.G.); alfonso.monaco@uniba.it (A.M.); ester.pantaleo@uniba.it (E.P.); tommaso.maggipinto@uniba.it (T.M.); antonio.lacalamita@uniba.it (A.L.C.); roberto.bellotti@uniba.it (R.B.); marianna.larocca@uniba.it (M.L.R.); 2 Istituto Nazionale di Fisica Nucleare, Sezione di Bari, 70125 Bari, Italy; loredana.bellantuono@uniba.it; 3 Dipartimento di Scienze del Farmaco, Università degli Studi di Bari Aldo Moro, 70125 Bari, Italy; 4 Perelman School of Medicine, University of Pennsylvania, Philadelphia, PA 19104, USA; kseniia.kriukova@pennmedicine.upenn.edu (K.K.); dominique.duncan@pennmedicine.upenn.edu (D.D.); 5 Dipartimento di Biomedicina Traslazionale e Neuroscienze, Università degli Studi di Bari Aldo Moro, 70124 Bari, Italy; 6 David Geffen School of Medicine at UCLA, Los Angeles, CA 90095, USA; pvespa@mednet.ucla.edu

**Keywords:** artificial intelligence, network modelling, diagnosis, image processing

## Abstract

Traumatic brain injury (TBI) is one of the leading causes of acquired epilepsy, with a significant proportion of patients developing post-traumatic epilepsy (PTE) even months or years after the initial injury. The identification of reliable imaging biomarkers able to predict epileptogenesis remains a major clinical challenge. In recent years, diffusion-weighted imaging (DWI) and structural connectome analysis have emerged as promising tools to investigate brain network alterations associated with late seizure development. Machine learning approaches may further support the detection of predictive patterns in complex neuroimaging data. The goal of this study is to perform a binary classification between seizure-free and late seizure-affected patients following TBI, with a specific focus on the identification of the anatomical regions potentially connected with late seizure development. A dataset of 59 diffusion weighted images (DWI) scans from the EpiBioS4Rx project, including 42 seizure-free and 17 late seizure-affected TBI patients, was analyzed. A Random Forest classification algorithm was applied, incorporating network feature importance based on the Gini index to investigate model’s decisions and allow a clinical interpretation. The model reported a 69% ± 0.03 accuracy for discrimination and a 73% AUC ± 0.05. Despite the limited and imbalanced nature of the dataset, and the fact that the performance does not significantly exceed chance once all data-dependent steps are taken into account, our approach allows us to achieve accurate classification results compared to the literature and to identify brain regions potentially associated with epileptogenesis.

## 1. Introduction

Traumatic brain injury (TBI) affects approximately 10 million individuals annually [1], representing one of the leading global causes of mortality and long-term disability. A critical complication of TBI is post-traumatic epilepsy (PTE), a neurological disorder characterized by recurrent, spontaneous seizures occurring at least one week after the initial injury. Due to the high inter-patient variability and the potentially long latency period often spanning several years between trauma and epilepsy onset, modeling and preventing epileptogenesis remains a major clinical challenge. The mechanisms underlying the development of PTE, particularly during the early post-injury stages, are still poorly understood [2]. TBI is a highly heterogeneous condition, with clinical outcomes strongly influenced by lesion type, size, and anatomical location. One of the most severe long-term complications of TBI is PTE, defined as epilepsy that develops following the injury. PTE is typically distinguished from seizures occurring in the acute phase after trauma, when the brain is still undergoing inflammatory and metabolic alterations. According to commonly adopted definitions, post-traumatic seizures can be classified into three temporal categories: immediate seizures, occurring within 24 h after injury; early seizures, occurring within the first week; and late seizures, occurring more than one week after trauma [3]. Because the risk of recurrence after a late post-traumatic seizure exceeds 70%, many investigators consider a single late seizure sufficient for the diagnosis of PTE [4].

Despite these clinical definitions, identifying reliable biomarkers capable of predicting epileptogenesis after TBI remains an open challenge.

In recent years, neuroimaging techniques have played a crucial role in the investigation of structural and functional brain alterations associated with TBI. In particular, diffusion-weighted imaging (DWI) and diffusion tensor imaging (DTI) allow the characterization of white matter structure and the reconstruction of structural brain networks. These techniques have shown that TBI can cause alterations in white matter pathways and brain connectivity, which may contribute to the development of epileptic activity. Network neuroscience approaches [5] based on graph-theoretical analysis have further improved the understanding of how brain connectivity changes after TBI.

Structural connectome analyses [6] allow researchers to study brain networks by describing how different regions are connected and how information flows across the network. Several studies suggest that changes in network organization may be related to the mechanisms underlying epileptogenesis and late seizure propagation [7].

In recent years, artificial intelligence techniques have rapidly expanded in the field of medical imaging, enabling the extraction of clinically relevant information from complex imaging datasets and supporting decision-making processes in several neurological and oncological applications [8]. In this context, machine learning (ML) approaches have been increasingly applied to neuroimaging data in order to identify predictive biomarkers of PTE and to better understand the mechanisms underlying epileptogenesis after traumatic brain injury. Several studies have explored the use of supervised learning algorithms to analyze structural and functional brain imaging features and to predict late seizure development in TBI patients [9]. For example, Akbar et al. demonstrated that machine learning models trained on diffusion MRI features can identify patterns associated with seizure susceptibility in TBI patients [10]. These results suggest that microstructural alterations detectable through diffusion-based connectivity analysis may provide useful predictive information for epileptogenesis [3].

Other studies have investigated the use of multimodal ML frameworks combining structural and functional imaging features. In particular, lesion normalization and supervised learning approaches have been proposed to improve seizure classification performance in TBI patients using diffusion MRI data [9].

Additional research has explored ML techniques applied to structural connectome features in order to characterize alterations in brain networks associated with neurological disorders. These approaches combine graph-theoretical descriptors of brain connectivity with classification algorithms to identify patterns associated with disease states [11].

However, many existing models mainly focus on prediction accuracy and provide limited interpretability, which reduces their usefulness in clinical practice. For this reason, explainable AI methods are increasingly being explored to improve the transparency and clinical relevance of ML models applied to neuroimaging data [12]. In addition, identification of structural connectivity alterations may potentially provide a relevant neurobiological foundation for future rehabilitation-oriented research: characterizing the topological reorganization of white matter networks following TBI may inform the design of targeted interventions aimed at restoring network integration in less severely affected populations [13].

The Epilepsy Bioinformatics Study for Anti-epileptogenic Therapy (EpiBioS4Rx) [14] is a large international multicenter project involving institutions such as UCLA, USC, the University of Eastern Finland, the University of Melbourne, and the Albert Einstein College of Medicine.

The project aims to collect longitudinal multimodal datasets in order to identify reliable biomarkers of epileptogenesis in both animal models and TBI patients, evaluate potential anti-epileptogenic therapies, and support the development of new treatment strategies. Within this framework, the present study aims to investigate structural brain connectivity alterations associated with post-traumatic epilepsy by combining DWI, structural connectome analysis, and machine learning techniques. In particular, we analyze graph-theoretical features derived from the structural connectome to characterize changes in brain network organization following traumatic brain injury. An explainable Random Forest classification model is then employed to discriminate between late seizure-affected and seizure-free patients and to highlight the most relevant connectivity features driving the classification. By integrating network neuroscience and interpretable machine learning, our goal is to identify brain regions and connectivity patterns that may serve as potential biomarkers of epileptogenesis in TBI patients.

## 2. Materials and Methods

### 2.1. Dataset Overview

**Figure 1:**
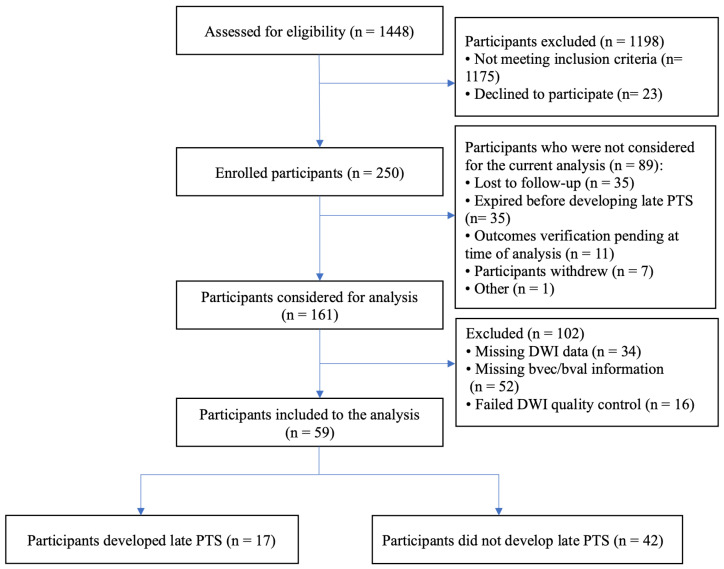
The CONSORT diagram shows that 1448 participants were assessed for eligibility, of whom 1198 were excluded, mainly for not meeting the inclusion criteria. A total of 250 participants were enrolled, but 89 were not considered for the final analysis due to loss to follow-up, death, or incomplete data. Among the 161 participants initially considered, further exclusions related to DWI data quality and completeness resulted in a final sample of 59 subjects. Of these, 17 developed late PTS, while 42 did not.

In this study, we used a dataset of 59 Diffusion Weighted Imaging (DWI) scans from the EpiBioS4Rx project, comprising 17 subjects who experienced at least one late seizure within 24 months of their TBI and 42 who remained seizure-free. DWI scans were collected with the following parameters: 64 directions, b-values = 0, 1000 s/mm^2^, minimum repetition time of 4000 ms, field-of-view = 256 mm, and slice thickness = 2 mm. The subject selection process is summarized in Figure 1. The full inclusion and exclusion criteria for the EpiBioS4Rx project are publicly available online (https://sites.google.com/g.ucla.edu/epibios4rxmobilewebsite/inclusionexclusion-criteria, accessed on 13 April 2026).

Patients were labeled as late seizure-affected if at least one late seizure episode was documented 1 week after TBI. The demographic and clinical data are summarized in Table 1. CT imaging data that were available are reported in Table 2.

Both groups include acute, sub-acute and chronic TBI and the distributions of the MRI post-injury days (see Figure S1 in the Supplementary Materials) are not different for the two groups at a 1% significance level using the Wilcoxon rank-sum statistic test.

### 2.2. Image Processing

Diffusion-Weighted Imaging (DWI) is an MRI-based technique that probes tissue microstructure by quantifying the displacement of water molecules. In intact neural tissue, water diffusion is highly organized and directionally constrained by axonal membranes; pathological processes such as inflammation, axonal injury, or gliosis disrupt this organization, resulting in measurable changes in diffusion parameters. This sensitivity to microstructural damage makes DWI a particularly valuable tool for detecting subtle cerebral alterations that may not be visible on standard structural imaging, including those potentially associated with seizure activity in the context of post-traumatic epilepsy.

Structural connectivity was reconstructed from DWI data using a widely adopted diffusion MRI processing pipeline implemented in MRtrix [15]. Preprocessing included eddy-current and motion correction, brain masking, and bias field correction. Fiber orientation distributions (FODs) were estimated using constrained spherical deconvolution [16], with the specific strategy adapted to single-shell or multi-shell acquisitions when appropriate.

For anatomical parcellation, the mean *b0* image was registered to the MNI152 template and the atlas was subsequently projected into the subject’s native diffusion space to define network nodes. Fiber tracking is the process by which white matter fiber bundle trajectories are reconstructed by propagating streamlines through the brain volume, step by step, following the dominant fiber orientations encoded in the FOD at each voxel. Whole-brain probabilistic tractography was performed using the iFOD2 algorithm, which samples candidate propagation directions probabilistically from the local FOD, rather than deterministically following its peak. This probabilistic framework makes the algorithm robust to noise and well-suited to resolve complex fiber configurations such as crossing and fanning fibers. Streamline weights were subsequently refined using SIFT2 to improve the quantitative correspondence between tractography density and the underlying white matter fibre distributions.

Structural connectomes were obtained by assigning reconstructed streamlines to atlas-defined regions and computing weighted connectivity matrices $W\in R^{96\times 96}$, where each element $w_{ij}$ represents the sum of SIFT2-weighted streamlines connecting regions *i* and *j* [17,18]. Default parameters of the MRtrix tractography pipeline were used; further methodological and implementation details can be found in the original references.

**Figure 2:**
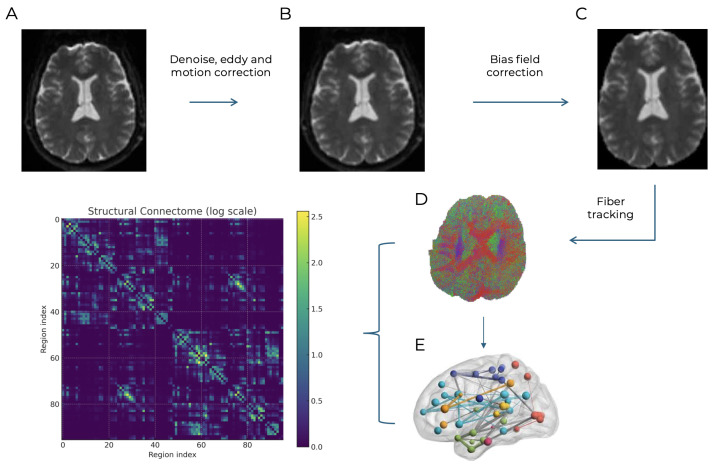
Diffusion MRI processing pipeline for structural connectivity reconstruction. (**A**) Raw DWI image. (**B**) Denoising, eddy-current and motion correction. (**C**) Bias field correction. (**D**) Probabilistic fiber tracking which is based on probabilistically sampling candidate propagation directions from the local fiber orientation distribution. (**E**) Construction of the weighted structural connectome, represented as a region-by-region connectivity matrix and corresponding brain network.

To account for inter-subject anatomical variability, the connectivity weights were normalized by the corresponding regional volumes. The schematic of the processing pipeline is reported in Figure 2.

### 2.3. Informative Network Features

Each structural connectivity matrix was modeled as a weighted undirected graph G=(V,E,W), where nodes (V) represent the brain regions defined by the Harvard–Oxford atlas, edges (E) represent the structural connections between pairs of regions, and edge weights (W) correspond to the normalized number of reconstructed streamlines connecting them. To assess the organization and informative content of the resulting brain networks, six weighted graph-theoretical metrics were evaluated as a function of network density [19]. To the best of our knowledge, there is currently no consensus or predefined density threshold in the specific context of post-traumatic epilepsy (PTE). Even in related fields (temporal lobe epilepsy or traumatic brain injury), studies typically adopt a range of densities rather than a single a priori threshold, often due to the lack of universally accepted standards [20,21].

#### 2.3.1. Integration and Segregation of the Brain Networks

Network integration, reflecting the efficiency of global information transfer, was quantified using the Average Shortest Path Length (ASPL), whereas network segregation, describing the tendency of nodes to form locally interconnected clusters, was assessed through the weighted clustering coefficient.

By defining the distance between nodes *i* and *j* as the reciprocal of the weight, $l_{ij}=1/w_{ij}$, the weighted Average Shortest Path Length of node *i* is defined as

(1) $$C_{\mathrm{ASPL}}\left(i\right)=\frac{1}{N-1}\sum_{j\neq i}d_{ij},$$

where $d_{ij}$ denotes the shortest weighted path length between nodes *i* and *j*.

The weighted clustering coefficient, describing the tendency of nodes to form locally interconnected groups, is defined as

(2) $$C_{\mathrm{cc}}^{w}\left(i\right)=\frac{1}{s_{i}\left(k_{i}-1\right)}\sum_{j\neq h}\frac{w_{ij}+w_{ih}}{2}\;a_{ij}a_{ih}a_{jh},$$

where $s_{i}=\sum_{j}w_{ij}$ is the strength of node *i*, $k_{i}$ its degree, and

$$a_{ij}=\left\{\begin{array}{cc} 1 & \mathrm{if}\;\mathrm{an}\;\mathrm{edge}\;\mathrm{between}\;i\;\mathrm{and}\;j\;\mathrm{exists},\\ 0 & \mathrm{otherwise}. \end{array}\right.$$

#### 2.3.2. Node Centrality Measures

To characterize the importance and influence of individual nodes within the network, strength, closeness centrality, betweenness centrality, and eigenvector centrality were computed.

The *strength* of node *i* is defined as

(3) $$C_{s}\left(i\right)=\sum_{j=1}^{N}w_{ij}.$$

The *closeness centrality*, measuring how efficiently a node communicates with the rest of the network, is defined as

(4) $$C_{c}\left(i\right)=\frac{N-1}{\sum_{j=1}^{N}d_{ij}}.$$

The *betweenness centrality*, quantifying the fraction of shortest paths passing through node *i*, is defined as

(5) $$C_{b}\left(i\right)=\frac{1}{\left(N-1)(N-2\right)}\sum_{\begin{array}{c} h\neq j\\ h\neq i,\;j\neq i \end{array}}\frac{\rho_{hj}\left(i\right)}{\rho_{hj}},$$

where $\rho_{hj}$ denotes the total number of shortest paths between nodes *h* and *j*, and $\rho_{hj}\left(i\right)$ the number of those paths passing through node *i*.

Finally, the *eigenvector centrality* assigns relative scores to nodes based on both the quantity and quality of their connections. It is defined as the leading eigenvector of the weighted adjacency matrix:

(6) $$x_{i}=\frac{1}{\lambda_{\max}}\sum_{j=1}^{N}w_{ij}x_{j},$$

where $\lambda_{\max}$ is the largest eigenvalue of the weighted adjacency matrix. The eigenvector centrality of node *i* is therefore defined as $C_{e}\left(i\right)=x_{i}$.

These metrics are widely used for the characterization of structural brain networks [22].

### 2.4. Global Network Analysis

As a first step, an exploratory analysis of the large-scale organization of the brain networks was performed to assess whether seizure-free and late seizure-affected patients exhibit systematic differences in their global topological properties. Mean weighted connectivity matrices were computed separately for seizure-free and late seizure-affected patients. Group-averaged connectomes were adopted to reduce inter-subject variability and improve the signal-to-noise ratio of connectivity estimates, as group-averaging partially mitigates the impact of spurious connections by retaining only pathways consistently observed across subjects [23]. For each density level, six weighted graph metrics were calculated on the group-averaged networks. Group differences were evaluated using the Wilcoxon rank-sum test, with statistical significance set at p<0.01. The analysis was repeated across the full range of network densities in order to assess the stability of the observed effects. It should be noted that network measures derived from group-averaged connectomes do not necessarily reflect the average of subject-level metrics, due to the nonlinear nature of graph-theoretical measures; therefore, the reported metrics should be interpreted as descriptors of group-representative topology rather than as summaries of inter-individual variability.

### 2.5. Local Selection and Density Range Selection

In graph-theoretical analysis of brain networks, density is defined as the ratio of existing connections (edges) to the maximum number of possible connections, expressed as a percentage. It controls the sparsity of the network: lower density values retain only the strongest connections, while higher values include progressively weaker ones.

Building on the insights provided by the global analysis, we then moved towards a more fine-grained characterization of the brain networks, focusing on local properties and node-level features.

**Figure 3:**
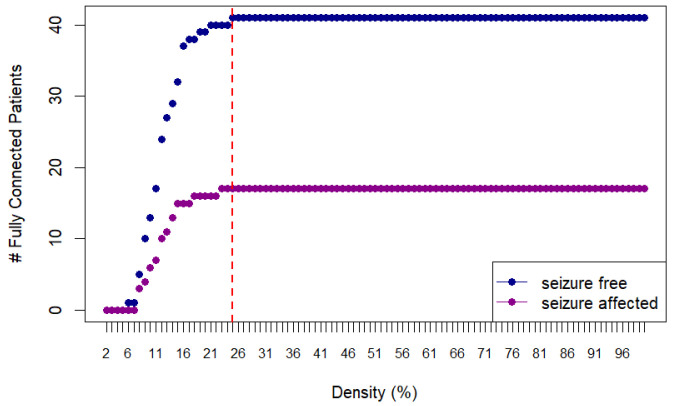
Number of fully connected brain networks across patients as a function of density. Full connectivity for the entire cohort is achieved at 25%.

First of all, to carry out the density study for the different network metrics, we selected the most appropriate density range. From a clinical perspective, a disconnected brain network lacks physiological interpretability, thus we defined the density at which all individuals’ brain networks became fully connected as the lower limit of the density range. A Breadth-First Search (BFS) [24] procedure was applied to each subject across increasing density levels. Figure 3 shows the number of fully connected brain networks as a function of density. As observed, full connectivity across the cohort is reached at 25%. Therefore, the lower threshold was set to 25%, ensuring both topological coherence and clinical interpretability.

**Figure 4:**
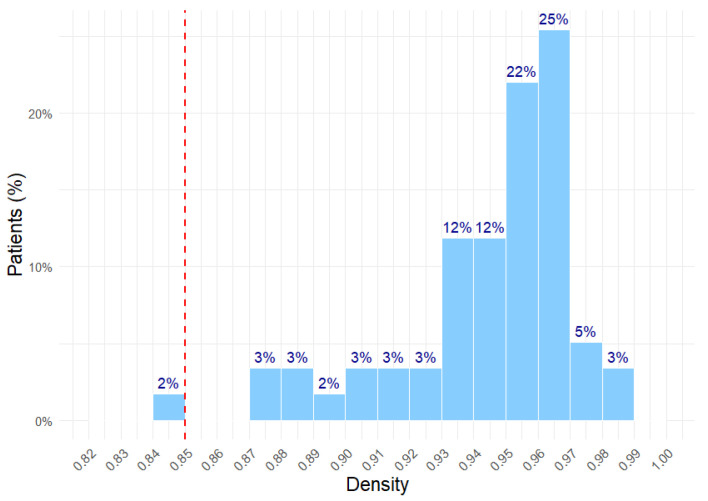
Histogram of the maximum achievable density for each subject. The upper bound was selected as the highest density (–85%) assignable to 98% of the cohort.

The upper density limit was defined to ensure that the same density could be applied across the brain networks of different subjects who initially exhibited varying baseline densities, thus a histogram of the maximum density reached by each subject was computed and the upper density threshold was defined as the value exceeded by 98% of the subjects. (see Figure 4).

The machine learning analysis was subsequently restricted to this density interval (25–85%), ensuring biological interpretability, topological validity, and methodological consistency across the cohort.

### 2.6. Machine Learning Workflow

For each density level, six weighted network metrics were computed across 96 brain regions, resulting in a total of 576 features per subject. Thirteen distinct datasets were generated by sampling the selected density range (25–85%) at 5% increments, ensuring adequate granularity while maintaining computational efficiency.

Classification performance was evaluated using 100 repeated cross-validation rounds with nested feature selection, where feature selection and Random Forest training were performed exclusively on the training data in each round to ensure robustness and prevent data leakage and overfitting. At each iteration, the dataset was randomly partitioned into a training set (90% of the data) and a test set (10% of the data). All preprocessing steps including centering and scaling, feature selection, and class balancing using SMOTE [22] were performed exclusively on the training data and subsequently training models were applied to the test set. Because repeated cross-validation folds are not statistically independent, variability across repetitions should be interpreted as a measure of stability rather than as a basis for parametric confidence intervals.

Feature selection was integrated within the classification pipeline using Gini importance scores derived from a Random Forest model [25]. First, feature relevance was assessed by computing the distribution of Gini importance values across all features. Features whose importance exceeded a threshold defined as the mean importance plus two standard deviations were identified as globally relevant predictors. Subsequently, for the final model construction, a minimum importance threshold of 0.01 was applied in order to retain only features contributing meaningfully to the classification task.

To assess feature stability and statistical relevance, the selection process was monitored across iterations by constructing a binary selection matrix indicating whether each feature exceeded the Gini threshold in a given round. Selection frequencies were compared against chance level using a binomial test, with Bonferroni correction applied for multiple comparisons (Significance level of 1% was used). For these features, the mean Gini importance across cross-validation rounds was computed to obtain a final ranking. This strategy was explicitly adopted to mitigate the instability and potential influence of the correlations on Gini individual importance estimates and to prioritize features that emerge robustly and consistently across multiple resamplings of the data.

Statistically significant and stable features were subsequently mapped onto the standard MNI152 brain template using MNI coordinates, providing anatomical interpretability of the results. Explainability analyses and clinical interpretation were performed using the network density level corresponding to the best classification performance.

## 3. Results

**Figure 5:**
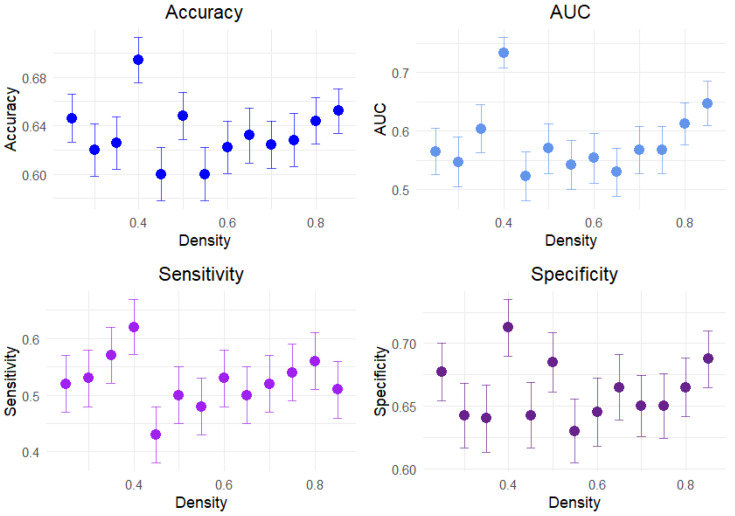
Classification performance (Accuracy, AUC, Sensitivity, Specificity) across network density thresholds ranging from 40% to 90%. Error bars represent variability across cross-validation folds. The 40% density threshold was selected for all subsequent analyses as it yielded the highest overall classification performance.

The study of the classification performances as a function of the different densities is reported in Figure 5. The best classification performance was achieved at 40% network density. Therefore, all subsequent analyses and discussion are based on results obtained at this density level.

The best classification results are summarized in Table 3. At this density level, the model achieved an accuracy of 0.69±0.03, with a sensitivity of 0.62±0.10 and a specificity of 0.71±0.05. The AUC reached 0.73±0.05, indicating moderate discriminative ability, consistent with the interpretation framework proposed by Hajian-Tilaki [26], whereby AUC values in the range 0.7–0.8 reflect acceptable diagnostic performance, meaningfully above chance level. Overall, the classifier demonstrated a balanced trade-off between sensitivity and specificity across the 100 cross-validation rounds.

**Figure 6:**
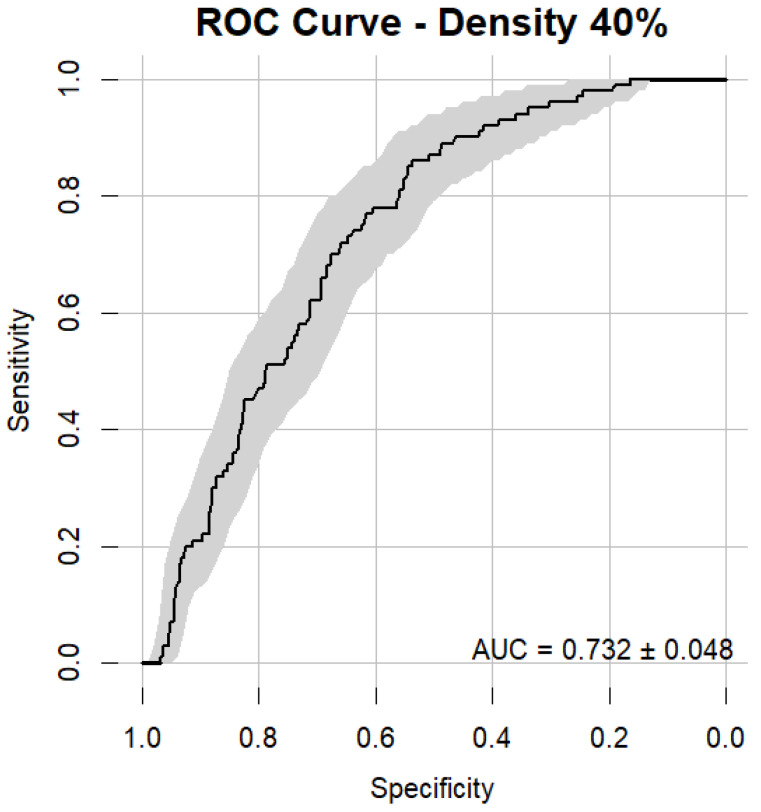
ROC curve at 40% network density averaged across cross-validation rounds. Shaded areas represent variability across folds.

The receiver operating characteristic (ROC) curve obtained at 40% network density is shown in Figure 6. The curve represents the mean ROC across cross-validation iterations, with shaded areas indicating the variability across folds.

**Figure 7:**
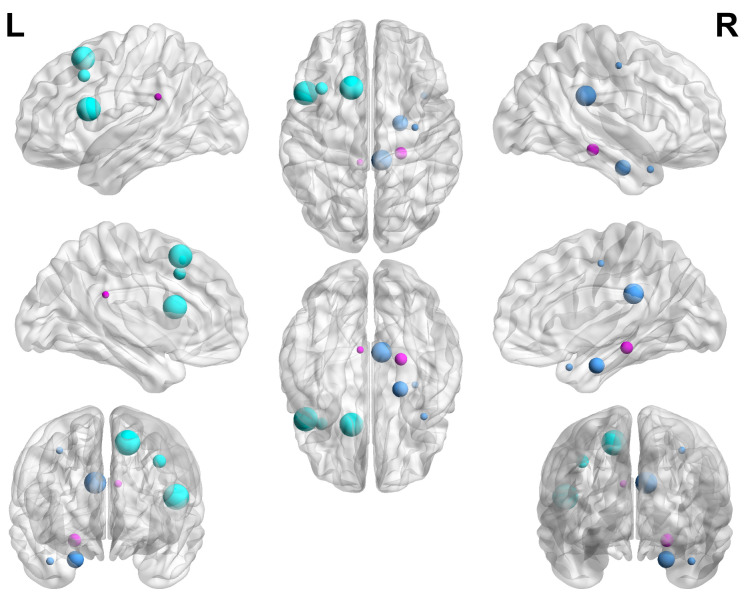
Brain Glass visualization of the most influential brain regions. Sphere size is proportional to feature importance, while colors represent different weighted network metrics (Eigenvector Centrality—light blue, Betweenness—dark blue, Clustering Coefficient—pink).

According to the embedded feature selection procedure and the subsequent binomial test with Bonferroni correction (α≤0.01), a total of 43 features were identified as statistically significant and stable across cross-validation iterations. To provide anatomical interpretability, the anatomical regions corresponding to the statistically significant and stable features were identified using the cortical Harvard–Oxford atlas. The visualization was performed using BrainNet Viewer [27], as shown in Figure 7. These features were distributed across four weighted network metrics: Betweenness, Clustering Coefficient, Eigenvector Centrality, and Strength.

**Figure 8:**
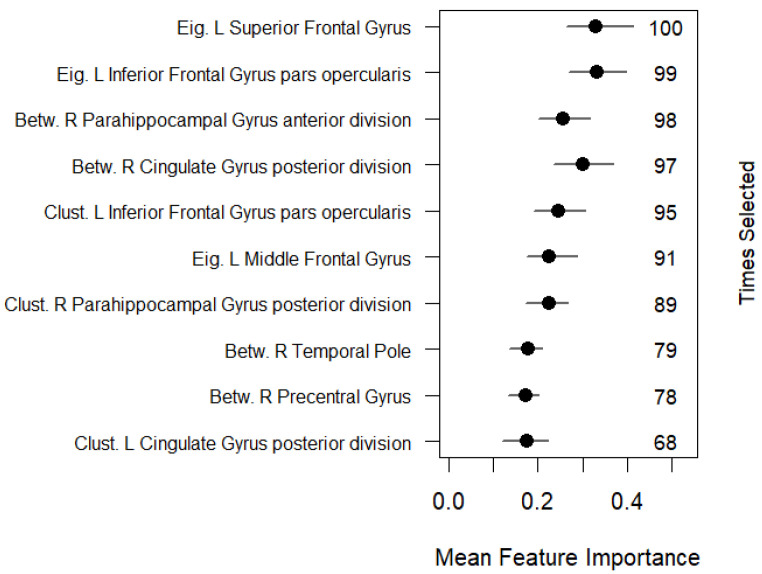
Top 10 most important features ranked by mean importance across 100 cross-validation rounds. Results are reported as median ± IQR.

The ten most influential features ranked by their average importance score across iterations are reported in Figure 8. Notably, regions within frontal, cingulate, and parahippocampal areas emerged as highly discriminative, suggesting a potential involvement of fronto-limbic network alterations in post-traumatic epileptogenesis. Additional regions beyond the top 10 are reported in the Supplementary Materials.

The selection of features across cross-validation rounds can be interpreted as a Bernoulli process, where each feature has a probability p of being selected at random. Under this assumption, the number of selections follows a binomial distribution, and deviations from the expected value np quantify the stability of the selection process [28]. To assess the stability of the features selected by the random forest model across cross-validation rounds, we computed a selection-based effect size defined as:

(7) $$d=\frac{n\;p(1-p)}{k-n\;p}$$

where *n* is the number of cross-validation rounds, *p* is the probability of a feature being selected by chance (1/total number of features), and *k* is the observed number of times the feature was selected.

This measure quantifies how consistently a feature is chosen relative to random expectation, with lower values indicating higher stability. Selection frequencies were used for statistical inference via binomial testing, while the selection-based effect size provides a descriptive measure of deviation from chance-level selection across cross-validation rounds, conceptually related to an unstandardized binomial z-score but intentionally avoiding variance-based standardization due to non-independence of cross-validation iterations. The results are reported in Table 4.

### Global Network Results

At the global level, among all evaluated metrics, significant differences between groups were observed exclusively for eigenvector centrality across densities ranging from 22% to 100%.

Specifically, seizure-free patients exhibited lower eigenvector centrality values compared to late seizure-affected patients. No other global metric showed statistically significant differences after thresholding.

## 4. Discussion

The machine learning framework, combined with the embedded feature selection procedure, identified eigenvector centrality as the most influential metric for distinguishing late seizure-affected from seizure-free patients. As shown in Figure 8, features derived from eigenvector centrality ranked among the most important predictors, highlighting a strong discriminative contribution of this measure at the regional level. To further investigate whether this local importance reflected broader network-level alterations, a complementary global network analysis was performed.

**Figure 9:**
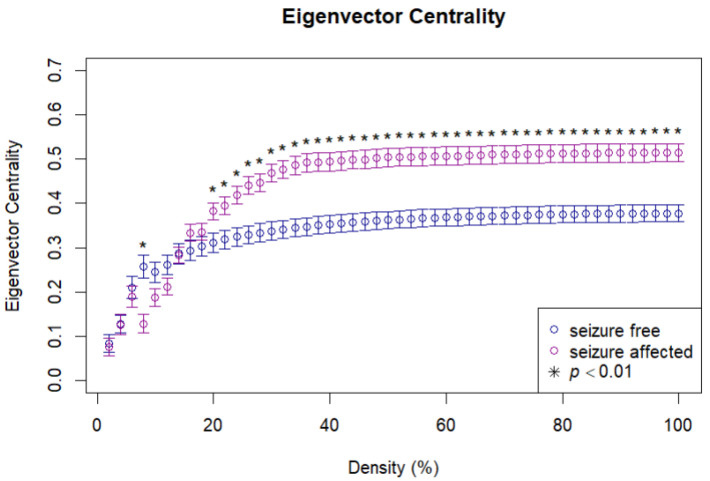
Eigenvector centrality as a function of network density for seizure-free TBI patients (blue) and late seizure-affected TBI patients (purple). Statistically significant differences between groups are indicated by stars (p<0.01).

As shown in Figure 9, eigenvector centrality was the only metric showing statistically significant differences between the two groups across a wide range of network densities. In particular, seizure-free patients exhibited consistently lower eigenvector centrality values compared with late seizure-affected patients.

The convergence between the local machine learning findings and the global graph-theoretical analysis suggests that alterations in hub-related network integration may represent a key characteristic of post-traumatic epileptogenesis.

Most prior studies analyzing neuroimaging features in TBI have focused on broader structural regions, and few have achieved gyrus-level resolution [29,30]. This limitation is largely attributable to the heterogeneity of TBI, and methodological constraints. The current approach, by contrast, enabled identification of more granular connectivity alterations, including discriminative features at the level of individual gyri.

The involvement of frontal regions is particularly coherent in this context. First, frontal lobe injury represents the most prevalent injury location in the study cohort, making it an anatomically expected locus of pathological reorganization. Second, frontal lobe lesions have been previously implicated in PTE [31], further supporting the biological plausibility of these findings at a finer anatomical scale. These findings provide a coherent network-level interpretation of the machine learning results and support the hypothesis that alterations in structural connectivity organization may contribute to the development of late seizures following traumatic brain injury.

Beyond the predictive accuracy of the model, the robustness of identified discriminative features is further supported by the selection-based effect size analysis. As shown in Table 4, the top ranked features in the superior and inferior frontal gyrus demonstrated remarkably low effect size values (ranging from 0.023 to 0.034). These values indicate a high degree of stability across cross-validation rounds, confirming that the importance of these nodal metrics is not an artifact of specific data partitioning but reflects consistent topological alterations within the TBI population. These findings should be contextualized within the constraints of an exploratory study; larger prospective cohorts will be necessary to establish whether these connectivity patterns generalize beyond the present sample.

Although direct comparisons with previous studies remain limited, our findings are broadly consistent with the emerging literature investigating connectivity alterations associated with post-traumatic epilepsy.

Akbar et al. [32] investigated the prediction of late seizure in traumatic brain injury patients using a multimodal framework that integrates features from 3 modalities: electroencephalography (EEG), resting state functional MRI (rs-fMRI) and dMRI. In their study, structural information was captured through fractional anisotropy (FA) derived from dMRI, electrophysiological markers were represented by the presence of epileptiform abnormalities found in EEG data, and functional alterations were characterized using fMRI based connectivity measures. The framework was evaluated on a cohort of 48 patients. The best performing model reported an AUC of 0.792, with sensitivity of 0.67 and specificity of 0.99 at the optimal operating point. In comparison, our current approach achieved a comparable AUC (0.73±0.05), with sensitivity of 0.62±0.10 and specificity of 0.71±0.05. This is reasonable considering that Akbar et al. [32] integrated multiple modalities and performed only a single round of 5-fold cross-validation. In the present study, a repeated random train-test splitting strategy was adopted: 100 independent splits were performed, such that each subject appeared in the test set multiple times across repetitions. Performance metrics were subsequently averaged across all repetitions, thereby reducing the variance associated with any individual data partition and providing a more stable estimate of out-of-sample performance. It should be acknowledged, however, that small test sets represent an inherent limitation of the available cohort size, and that performance estimates should be interpreted accordingly. Indeed, while cross-validated performance suggests moderate discriminative ability, permutation testing of the full analysis pipeline indicates that this performance does not significantly exceed chance once all data-dependent steps are taken into account (for further details see Figure S6 in the Supplementary Materials). These results highlight the necessity of permutation-based inference when complex pipelines are employed and suggest caution when interpreting cross-validated performance estimates in small or imbalanced datasets.

Awan et al. [33] developed predictive models to estimate the individual risk of epilepsy within two years following TBI. Their study included 6089 participants, of whom 4126 had complete late seizure follow-up data over the two-year period. Using regularized logistic regression models based on clinical and demographic predictors, the authors reported predictive performances with an AUC ranging between 0.67 and 0.84, depending on the specific model configuration and prediction task. These performances are comparable with those obtained in the present study, despite methodological differences and the considerably smaller dataset used in our analysis.

While clinical and demographic models, such as those proposed by Awan et al., provide valuable risk stratification, they do not account for the underlying neurobiological reorganization of the brain. A network neuroscience perspective, however, offers a powerful framework to investigate. Brain connectivity models have shown that both local and global topological properties can reveal patterns associated with neurological disorders [34]. In line with this perspective, recent studies employing graph-theoretical analysis of functional connectivity have reported that TBI patients who develop late seizures exhibit disrupted network organization. Specifically, these networks appear more hyperconnected and hyperintegrated, while simultaneously showing reduced segregation and alterations in hub-related properties [35]. Notably, these studies reported a higher number of low-betweenness hubs in patients who developed late seizures compared with seizure-free individuals, suggesting that hub-related network organization may play a role in post-traumatic epileptogenesis. Within this context, eigenvector centrality represents a measure of node influence by assigning higher scores to nodes that are connected to other highly connected nodes in the network. Alterations in this metric may therefore indicate a pathological shift in hub-mediated integration. Nevertheless, given the limited number of studies specifically addressing post-traumatic epilepsy using structural connectome analysis, direct comparisons remain difficult. It is worthwhile to note that our findings should be considered preliminary rather than definitive, given the relatively limited sample size. Future studies on larger, independent cohorts will be essential to confirm the robustness and generalizability of these results. Several factors likely contribute to the modest classification accuracy. The limited sample size and class imbalance (42 seizure-free vs. 17 late seizure-affected patients) constrain statistical power and generalization. TBI’s inherent heterogeneity in injury mechanism, severity, and anatomical distribution, introduces biological noise that structural connectome features alone may not fully capture. Additionally, diffusion-weighted tractography carries well-known limitations regarding false-positive and false-negative tract reconstructions, and variability in post-injury imaging time points across subjects may reflect different stages of the epileptogenic process, introducing temporal confounds not accounted for within a binary classification framework. It should be noted that post-traumatic edema and neuroinflammatory processes may increase extracellular free water and influence diffusion-derived connectivity measures. While free-water-related effects are often considered a confounding factor for accurate white matter microstructural estimation, they may also represent biologically meaningful features associated with epileptogenesis after TBI. However, since no dedicated free-water modeling approach was applied, the present findings cannot fully disentangle alterations related to tissue microstructure from those associated with extracellular inflammatory changes. Besides, in the context of post-traumatic epilepsy, impaired ARC-mediated homeostatic regulation may contribute to network hyperexcitability and synaptic remodeling. Crucially, the downstream consequences of this dysregulation (including axonal injury, membrane disruption, and neuroinflammatory cascades) are precisely the microstructural processes detectable by DWI [36]. In the future, if also cytokines and ARC protein concentrations are available, it could be interesting to study the interplay of diffusion-based connectivity alterations and neuroinflammatory mechanisms following TBI.

## 5. Conclusions

This study proposed a machine learning framework based on structural connectome analysis combined with embedded feature selection to investigate biomarkers associated with late-seizure development following TBI. Our results identified eigenvector centrality as the most relevant feature for distinguishing seizure-free from late seizure-affected patients, consistently emerging from both local feature importance analysis and global network comparisons. This suggests that alterations in hub-related network integration represent a key characteristic of post-traumatic epileptogenesis. Importantly, the proposed approach not only provides predictive performance but also enables the identification of the brain regions and connectivity metrics most strongly associated with the disease. Notably, the regions identified in this study are in agreement with existing literature, further supporting the biological plausibility of the proposed framework and its potential to capture meaningful patterns of network reorganization. The model achieved an accuracy of 0.69±0.03, with a sensitivity of 0.62±0.10, specificity of 0.71±0.05, and an AUC of 0.73±0.05. Further studies on larger cohorts are needed to validate these findings and strengthen their clinical applicability.

## Figures and Tables

**Table 1:** Demographic and clinical summary of patients included in the connectome analysis, stratified by seizure outcome.

Clinical Status	Sample Size	Age	Female/Male	GCS Score (Day 1)
Seizure-free patients	42	48.26±20.63	11/31	7.46±3.34
Patients with late seizure	17	43.12±17.27	2/15	7.59±3.06

**Table 2:** Clinical and CT information for labeled patients (N = 59), stratified by seizure outcome. Percentages are computed within each outcome group over the full cohort (N = 59).

Variable	All (N = 59)	No Seizure (N = 42)	Seizure (N = 17)
*Injury Type* ^†^			
Closed	55/59 (93%)	40/42 (95%)	15/17 (88%)
Penetrating	1/59 (2%)	1/42 (2%)	0/17 (0%)
*Injury Mechanism (grouped)*			
Motor vehicle (incl. motorcycle)	31/59 (53%)	22/42 (52%)	9/17 (53%)
Fall	14/59 (24%)	8/42 (19%)	6/17 (35%)
Direct impact	6/59 (10%)	4/42 (10%)	2/17 (12%)
Cycling/micromobility	5/59 (8%)	5/42 (12%)	0/17 (0%)
Other	3/59 (5%)	3/42 (7%)	0/17 (0%)
*CT Findings* ^‡^			
Skull fracture	23/59 (39%)	11/42 (26%)	12/17 (71%)
Subarachnoid hemorrhage	23/59 (39%)	11/42 (26%)	12/17 (71%)
Intracerebral hemorrhage	20/59 (34%)	12/42 (29%)	8/17 (47%)
Hemorrhagic contusion	21/59 (36%)	13/42 (31%)	8/17 (47%)
Intraventricular hemorrhage	3/59 (5%)	2/42 (5%)	1/17 (6%)

^†^ 3 patients have no injury type recorded. ^‡^ CT hemorrhagic information available for 23/59 patients.

**Table 3:** Classification performance at 40% network density. Results are reported as mean ± standard deviation across 100 cross-validation rounds.

Metric	Mean ± SD	95% CI
Accuracy	0.69±0.03	[0.66,0.73]
Sensitivity	0.62±0.10	[0.52,0.72]
Specificity	0.71±0.05	[0.67,0.76]
AUC	0.73±0.05	[0.68,0.79]

**Table 4:** Top graph features selected by the model with corresponding selection effect size across cross-validation rounds.

Feature	Effect Size
Eig. L Superior Frontal Gyrus	0.023
Eig. L Inferior Frontal Gyrus pars opercularis	0.023
Betw. R Cingulate Gyrus posterior division	0.023
Betw. R Parahippocampal Gyrus anterior division	0.023
Clust. L Inferior Frontal Gyrus pars opercularis	0.024
Clust. R Parahippocampal Gyrus posterior division	0.025
Eig. L Middle Frontal Gyrus	0.025
Clust. L Cingulate Gyrus posterior division	0.030
Betw. R Precentral Gyrus	0.030
Betw. R Temporal Pole	0.034

## Data Availability

The data analyzed in this study is subject to the following licenses/restrictions: access to data must be requested and approved by the EpiBioS4Rx steering committee. Requests to access these datasets should be directed to epibiossteeringcommittee@loni.usc.edu.

## Supplementary Materials

The following supporting information can be downloaded at: https://www.mdpi.com/article/10.3390/bioengineering13060598/s1, Figure S1: Histogram of the MRI post-injury days for the seizure-affected subjects (blue bins) and the seizure-free subjects (red bins); Figure S2: Spatial distribution of regions identified using betweenness centrality; Figure S3: Spatial distribution of regions identified using strength; Figure S4: Spatial distribution of regions identified using the clustering coefficient; Figure S5: Spatial distribution of regions identified using eigenvector centrality; Figure S6: Permutation test results showing the AUC distribution obtained by randomly permuting class labels across 1000 iterations; Table S1: Total GCS scores (mean ± SD) from Day 2 to Day 14, stratified by seizure outcome; Table S2: Feature importance ranking based on mean Gini importance across cross-validation runs.
