# Supplementary material for: A Brain Connectivity Approach to Detect Diffusion-Weighted Imaging Changes in Post-Traumatic Epilepsy
Source: Bioengineering (Basel). 2026 May 22;13(6):598. doi: 10.3390/bioengineering13060598 (PMC13296116; doi:10.3390/bioengineering13060598)
Supplement: Supplementary file 1 [file bioengineering-13-00598-s001.zip › bioengineering-4286694-supplementary.pdf]

Supplementary Materials

1 GCS Scores

Table 1: Total GCS scores (mean ± SD) from Day 2 to Day 14, stratified by seizure outcome.

**GCS**	**Seizure-free** ($n = 42$)	**Late seizure** ($n = 17$)
Day 2	7.96 ± 3.16	6.70 ± 2.53
Day 3	7.74 ± 3.48	6.47 ± 2.40
Day 4	8.29 ± 3.97	5.71 ± 2.41
Day 5	8.52 ± 4.12	5.94 ± 3.09
Day 6	8.70 ± 4.18	6.24 ± 2.95
Day 7	8.39 ± 4.15	6.89 ± 3.08
Day 14	10.74 ± 4.23	8.12 ± 3.79

2 Density study

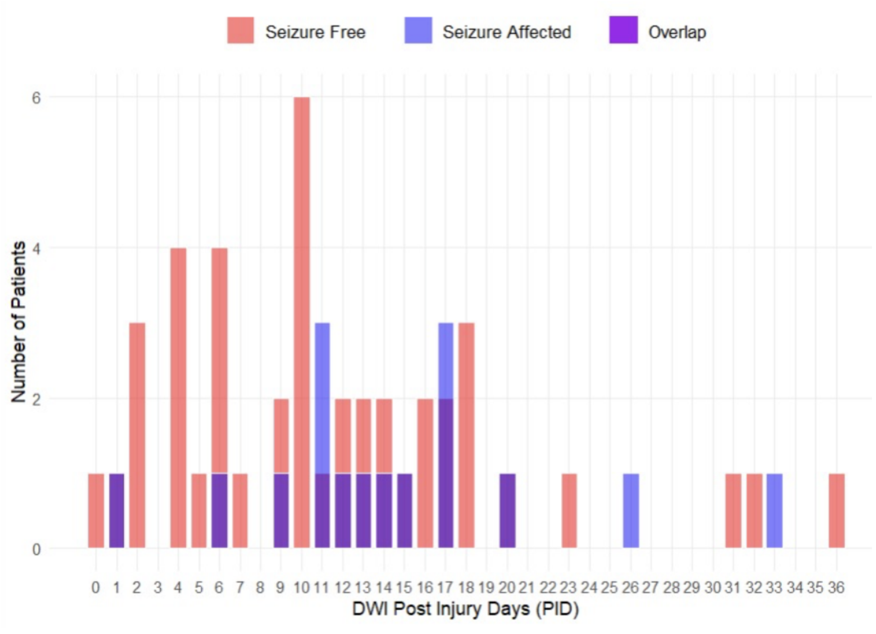

Figure S1: Histogram of the MRI post-injury days for the seizure-affected subjects (blue bins) and the seizure-free subjects (red bins). Purple bins represent the overlap between the two groups. Both groups include subjects with acute, subacute, and chronic TBIs. The distribution of MRI post-injury days is not significantly different between the two groups ($p < 0.01$).

3 Spatial Distribution of Discriminative Brain Regions

This section illustrates the spatial distribution of brain regions identified as relevant by each network metric. Node size reflects feature importance derived from the classification model.

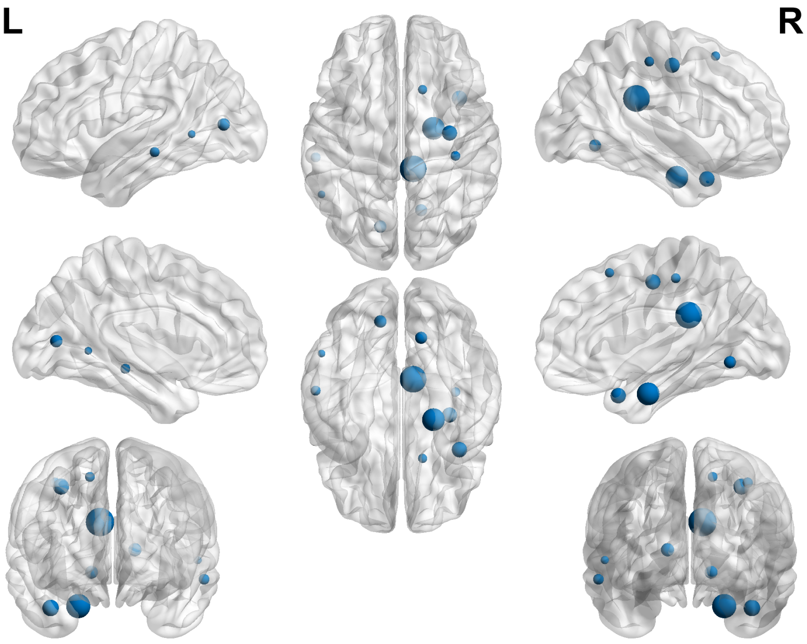

Figure S2: Spatial distribution of regions identified using betweenness centrality.

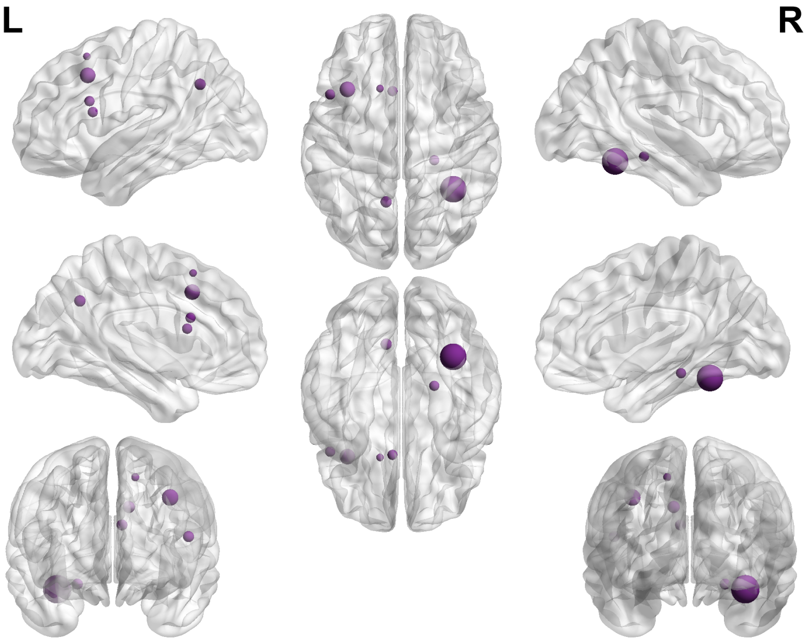

Figure S3: Spatial distribution of regions identified using strength.

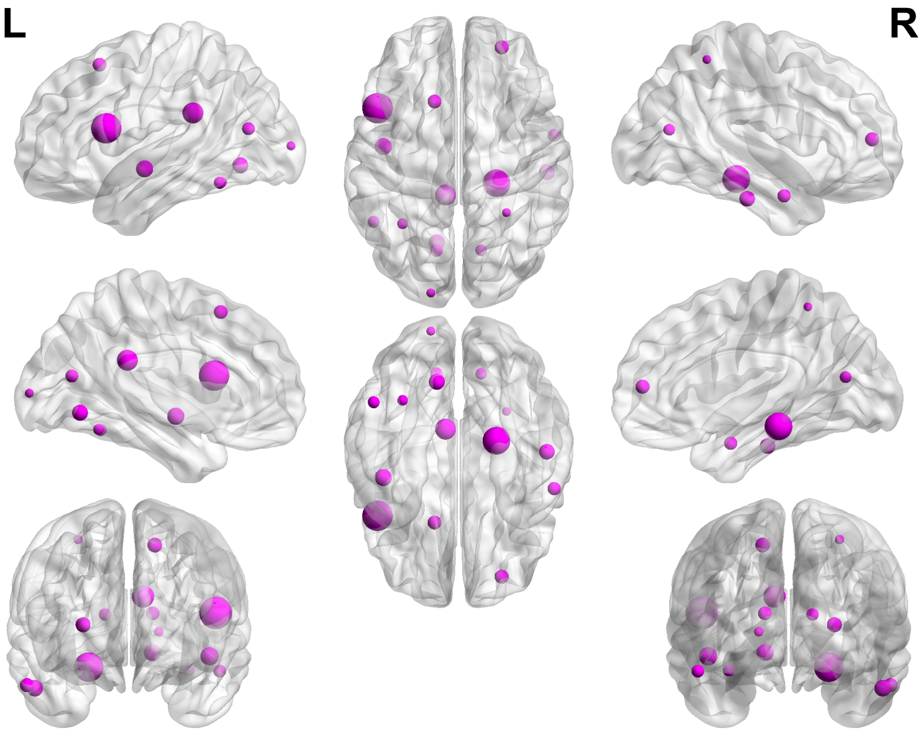

Figure S4: Spatial distribution of regions identified using the clustering coefficient.

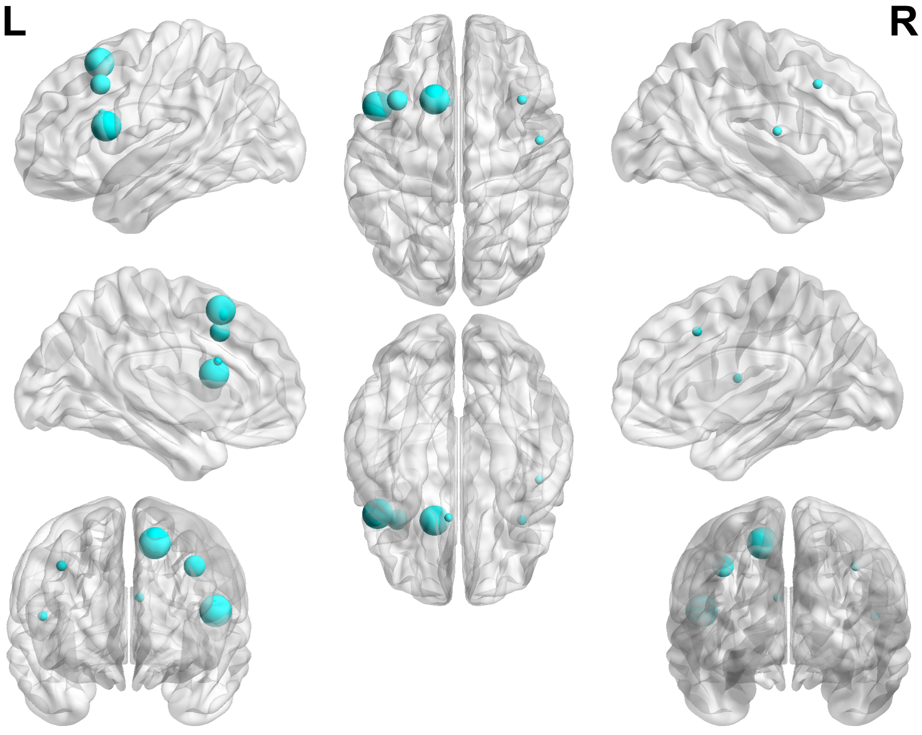

Figure S5: Spatial distribution of regions identified using eigenvector centrality.

4 Permutation study

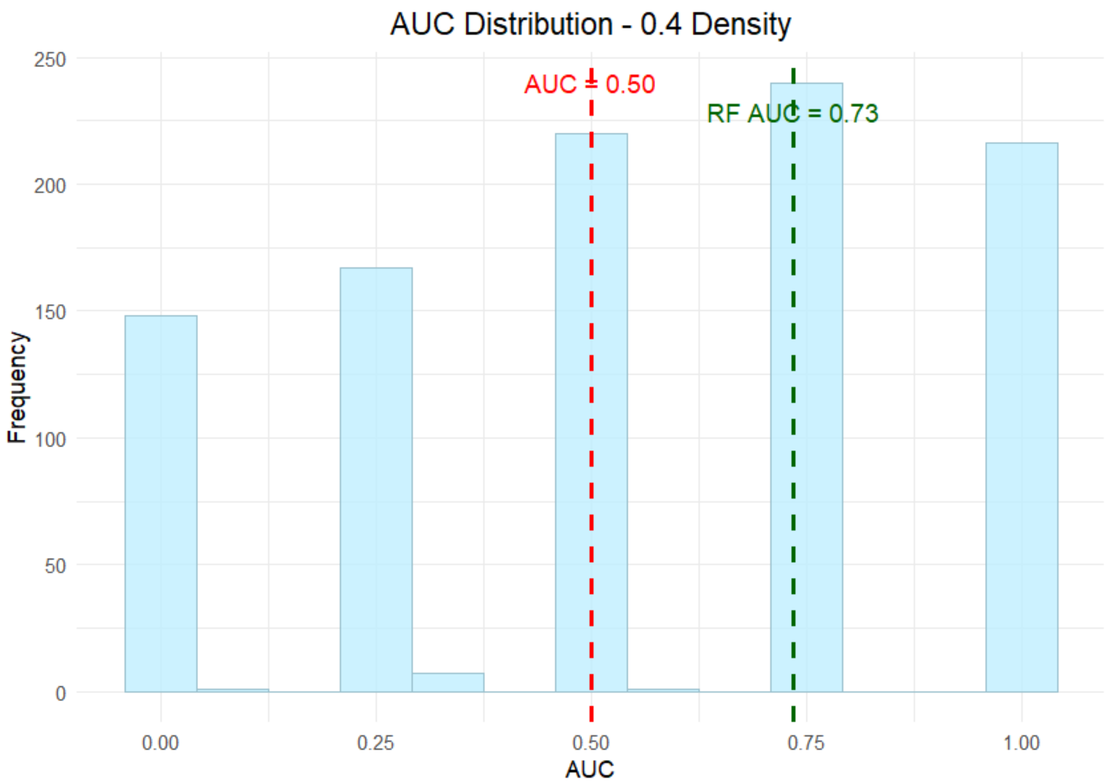

Figure S6: Permutation test results. AUC distribution obtained by randomly permuting class labels across 1000 iterations while keeping the full pipeline intact (feature selection, SMOTE, density-based selection, and model training). The red dashed line indicates chance-level performance (AUC = 0.5), while the green dashed line marks the observed AUC (0.73). The observed AUC falls within the permutation distribution and does not appear as an outlier.

5 Feature Importance Ranking

The table below reports the most relevant features ranked according to their mean Gini importance across cross-validation runs.

Feature	Importance
Eigenvector Left Inferior Frontal Gyrus (pars opercularis)	0.333
Eigenvector Left Superior Frontal Gyrus	0.330
Betweenness Right Posterior Cingulate Gyrus	0.300
Betweenness Right Anterior Parahippocampal Gyrus	0.257
Clustering Left Inferior Frontal Gyrus (pars opercularis)	0.245
Clustering Right Posterior Parahippocampal Gyrus	0.225
Eigenvector Left Middle Frontal Gyrus	0.225
Betweenness Right Temporal Pole	0.178
Clustering Left Posterior Cingulate Gyrus	0.176
Betweenness Right Precentral Gyrus	0.174
Clustering Left Planum Polare	0.149
Betweenness Left Intracalcarine Cortex	0.145
Strength Right Temporal Occipital Fusiform Cortex	0.145
Betweenness Right Lingual Gyrus	0.141
Clustering Left Lingual Gyrus	0.141
Clustering Right Inferior Temporal Gyrus (posterior division)	0.138
Clustering Right Frontal Pole	0.127
Clustering Left Superior Frontal Gyrus	0.125
Strength Left Juxtapositional Lobule Cortex (SMA)	0.125
Clustering Right Middle Temporal Gyrus (anterior division)	0.121
Strength Left Middle Frontal Gyrus	0.121
Betweenness Left Middle Temporal Gyrus (posterior division)	0.118
Clustering Left Supracalcarine Cortex	0.118
Betweenness Right Postcentral Gyrus	0.116
Strength Left Precuneous Cortex	0.112
Eigenvector Right Middle Frontal Gyrus	0.112
Clustering Left Inferior Temporal Gyrus (temporo-occipital)	0.112
Strength Right Heschl's Gyrus	0.112
Betweenness Right Superior Frontal Gyrus	0.111
Strength Left Inferior Frontal Gyrus (pars opercularis)	0.110
Strength Left Anterior Cingulate Gyrus	0.110
Strength Right Posterior Parahippocampal Gyrus	0.110
Clustering Right Supracalcarine Cortex	0.109
Eigenvector Right Central Opercular Cortex	0.106
Strength Left Superior Frontal Gyrus	0.104
Clustering Left Temporal Occipital Fusiform Cortex	0.103
Clustering Left Heschl's Gyrus	0.098
Eigenvector Left Anterior Cingulate Gyrus	0.097
Strength Right Parietal Operculum Cortex	0.096
Betweenness Left Middle Temporal Gyrus (temporo-occipital)	0.093
Clustering Right Juxtapositional Lobule Cortex (SMA)	0.091
Clustering Left Occipital Pole	0.089
Clustering Right Superior Parietal Lobule	0.088

Table 2: Feature importance ranking based on mean Gini importance across cross-validation runs.
